# BarleyVarDB: a database of barley genomic variation

**DOI:** 10.1093/database/baaa091

**Published:** 2020-11-28

**Authors:** Cong Tan, Brett Chapman, Penghao Wang, Qisen Zhang, Gaofeng Zhou, Xiao-qi Zhang, Roberto A Barrero, Matthew I Bellgard, Chengdao Li

**Affiliations:** Western Barley Genetics Alliance, Agricultural Sciences, College of Science, Health, Engineering and Education, Murdoch University, 90 South Street, Murdoch, WA 6150, Australia; Western Barley Genetics Alliance, Agricultural Sciences, College of Science, Health, Engineering and Education, Murdoch University, 90 South Street, Murdoch, WA 6150, Australia; Western Barley Genetics Alliance, Agricultural Sciences, College of Science, Health, Engineering and Education, Murdoch University, 90 South Street, Murdoch, WA 6150, Australia; Australian Export Grains Innovation Centre, 3 Baron-Hay Court, South Perth, WA6151, Australia; Department of Primary Industries and Regional Development, Government of Western Australia, 3 Baron-Hay Court, South Perth, WA 6151, Australia; Western Barley Genetics Alliance, Agricultural Sciences, College of Science, Health, Engineering and Education, Murdoch University, 90 South Street, Murdoch, WA 6150, Australia; eResearch Office, Queensland University of Technology, 2 George St, Brisbane, QLD 4001, Australia; eResearch Office, Queensland University of Technology, 2 George St, Brisbane, QLD 4001, Australia; Western Barley Genetics Alliance, Agricultural Sciences, College of Science, Health, Engineering and Education, Murdoch University, 90 South Street, Murdoch, WA 6150, Australia; Department of Primary Industries and Regional Development, Government of Western Australia, 3 Baron-Hay Court, South Perth, WA 6151, Australia

## Abstract

Barley (*Hordeum vulgare L.*) is one of the first domesticated grain crops and represents the fourth most important cereal source for human and animal consumption. BarleyVarDB is a database of barley genomic variation. It can be publicly accessible through the website at http://146.118.64.11/BarleyVar. This database mainly provides three sets of information. First, there are 57 754 224 single nuclear polymorphisms (SNPs) and 3 600 663 insertions or deletions (InDels) included in BarleyVarDB, which were identified from high-coverage whole genome sequencing of 21 barley germplasm, including 8 wild barley accessions from 3 barley evolutionary original centers and 13 barley landraces from different continents. Second, it uses the latest barley genome reference and its annotation information publicly accessible, which has been achieved by the International Barley Genome Sequencing Consortium (IBSC). Third, 522 212 whole genome-wide microsatellites/simple sequence repeats (SSRs) were also included in this database, which were identified in the reference barley pseudo-molecular genome sequence. Additionally, several useful web-based applications are provided including JBrowse, BLAST and Primer3. Users can design PCR primers to asses polymorphic variants deposited in this database and use a user-friendly interface for accessing the barley reference genome. We envisage that the BarleyVarDB will benefit the barley genetic research community by providing access to all publicly available barley genomic variation information and barley reference genome as well as providing them with an ultra-high density of SNP and InDel markers for molecular breeding and identification of functional genes with important agronomic traits in barley.

**Database URL**: http://146.118.64.11/BarleyVar

## Background

Single nuclear polymorphisms (SNPs) and insertions or deletions (InDels) are the two most common types of genetic variations among living organisms. They have played essential roles in examining genetic diversity (1), positional cloning (2–4), association mapping (5–7) and evolutionary biology (8, 9) in the past decades. Barley (*Hordeum vulgare L.*) is one of the first domesticated grain crops (10, 11) and has been the fourth most important cereal source for human and animal consumption. It is widely used as a model organism to research the genetic basis of plant adaptive evolutionary processes and as a vital genetic repository to explore plant abiotic and biotic stress tolerance, as it can be grown in diverse and extreme environments such as warm-dry Near East and in cold-dry Tibet (12). The availability of the barley reference genome achieved this year (13), and the dramatic advantage of next-generation sequencing technology will make it more efficient to explore genomic variation in barley germplasm, which will accelerate barley genomic and genetic research progress. There are several genomic variation databases such as RiceVarMap (14), SNP-Seek (15) and HapRice (16) conducted in rice and dbSNP in the National Center for Biotechnology Information (NCBI) (17). There however, has not been any public database available of genomic variation information in barley until recently, except several databases for molecular markers such as GrainGenes (18) and barley physical map information of Barlex (19). At this stage, a comprehensive database of barley genomic variation is in an urgent need for the barley genomic research community to share and make good use of the genomic variation information exploited by different research groups. Here, we constructed a comprehensive database specializing in barley genomic variation and designated it as BarleyVarDB.

In summary, the BarleyVarDB database mainly includes three sets of data. First, 57 754 224 SNPs and 3 600 663 InDels identified from high coverage whole genome sequencing (∼40X) of 20 diverse barley germplasms representing wild barley accessions from 3 widely accepted barley evolutionary original centers and cultivar barley accessions from different continents. Second, it makes the latest barley genome reference and corresponding gene annotation information accessible to the research public, a genomic resource generated by the International Barley Genome Sequencing Consortium (IBSC) (13). Third, 522 212 microsatellites, also called simple sequence repeats (SSRs), are included in this database and identified by assessing the current barley pseudo-molecular genome sequence. In addition, BarleyVarDB provides several user-friendly web-based tools such as the genetic genome browser (JBrowse) (20) for displaying genetic information at genome or chromosome wide scale, basic local alignment search tool (BLAST) (21) for mapping query sequences against the barley genome and annotated sequences, and primer3 (22) for primer design. BarleyVarDB is publicly accessible through the website interface at http://146.118.64.11/BarleyVar.

### Database construction and content

**Figure 1.**
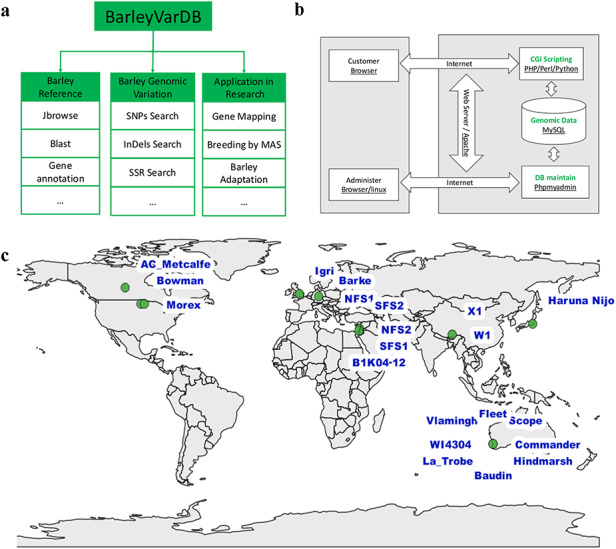
Framework, implementation, and date collection of BarleyVarDB. (a) The framework and aims for building BarleyVarDB. (b) The overview of the implementation of BarleyVarDB. (c) The geographic distribution map of barley accessions collected in BarleyVarDB.

**Figure 2.**
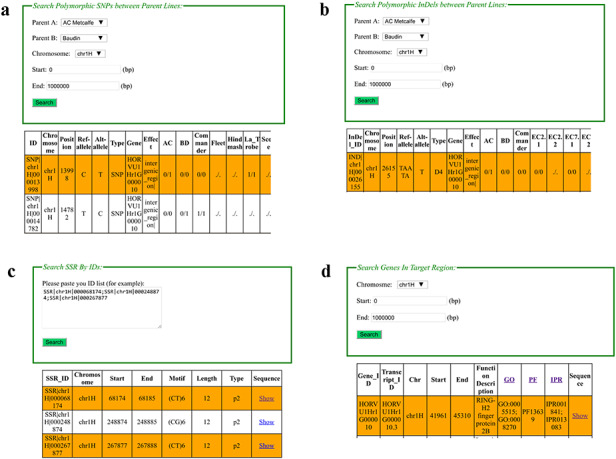
Web interfaces of retrieving SNPs, InDels, SSRs and gene annotation in BarleyVarDB. (a) Main interface of SNP search input and result output. (b) Main interface of InDel search input and result output. (c) Main interface of SSRs search input and result output. (d) Main interface of gene annotation search input and result output.

The structure of the BarleyVarDB is depicted on Figure 1a. The first aim of this database is to share and make full use of the genomic variants (SNPs, InDels) mined from increasingly accumulated genome sequencing data in barely research community. These genomic variants can be retrieved by different query keywords such as variants identifier, target region, candidate gene and so on (Figure 2). The second aim is to distribute the updated barley genome reference with high quality to the public and also make it easily accessible to the barley genetic researcher without programming experience through web-based applications, namely, JBrowse and BLAST. Both the genomic variants and barley genome reference pave the way for gene mapping of important agronomic traits, new competitive varieties breeding using molecular marker assisted selection and research into the adaptive evolution and domestication of barley.

BarleyVarDB was mainly implemented using the open-source HTTP server of Apache2 and the relational database management system of MySQL (Figure 1b). Apache servers provide the web interface for database clients to visit and interact with the database server using internet browsers. All the genomic variations and barley reference genome annotation information are stored and maintained in a MySQL database server. Clients can retrieve information through an internet browser and Apache server. Genomic variants database can be maintained and updated using MySQL server commands in Linux or using phpMyAdmin through web browser. Also, custom Perl, python and PHP scripts were used to display the results in a user-friendly format and to enable basic functions such as keywords searching for multiple queries.

At present, we have collected ∼3.70 Tbs sequencing data of whole genome sequencing of 21 barley accessions in high coverage (Table 1), which represent wild barley from 3 evolution centers and cultivar barley from different continents (Figure 1c). The first dataset comprises of six wild barley accessions from the Tibetan plateau and opposing slopes of ‘Evolution Canyon’ as well as nine cultivar barley accessions from Australia and Canada. The detailed description of sample collection and whole genome sequencing for this dataset were given in another paper (23). In brief, these barley germplasms were planted in a Murdoch University glass house and genomic DNA was extracted from leaves of the plants at three leaf stage. Sequencing library for each sample was prepared following the manufacturer’s instruction (Paired-End Sample Preparation Guide, Illumina, 1 005 063) and then sequenced on the Illumina HiSeq 2000 platform in BGI-Shenzhen (Beijing Genomics Institute-Shenzhen, Shenzhen, China). Eventually, ∼2.96 Tb high quality reads were produced from these 15 barley accessions and the raw data submitted to NCBI/SRA with BioProject accession number PRJNA324520. The second dataset (∼736 Gbs) comprises of five barley cultivars and one wild barley accession (24), which was performed by Leibniz Institute of Plant Genetics and Crop Plant Research (IPK). It was downloaded from European Nucleotide Archive (ENA, EMBL-EBI) with the accession number PRJEB628.

The detailed methodology for mapping reads against reference and genomic variation detection for the collected sequencing data can refer to our published paper (23), and major scripts used in the data processing of this project was given in our Github site, and detailed description of methods producing genomic variants shared in BarleyVarDB was provided in the supplementary note page. The summary is given as follows. First, a strict filtration was performed to remove reads with contamination and trim poor-quality bases for each accession. Then, the barley pseudo-molecular genome was used as the reference to mapping the clean reads using BWA-MEM (25). On average, ∼98% of clean reads were mapped to the reference sequence and ∼49% of them have unique mapping positions in the reference with high mapping quality. Next, only those having unique mapping positions remained and proceeded to detect genomic variation (SNPs and InDels). The final SNPs and InDels were generated by running two rounds of variants calling analysis using SAMtools/BCFtools (26, 27) and GATK (28) pipelines. The first round was performed with SAMtools and BCFtools for all accessions and the result was controlled based on the variants’ quality. Then, the calling result of the first round was used as the input file to guide the realignment around potential InDels and variants calling for the second round in GATK. The SNPs and InDels were detected by both SAMtools/BCFtools pipeline and GATK were retained and then strictly filtered mainly based on base quality, mapping quality and variants supporting depth (29). The accuracy of SNP calling was up to 98.95% and validated by comparing the sequence difference detected by pairwise alignment between the de novel assembly contig Bowman_contig_843756 (34 593 bp) and its corresponding genomic region chr3H:419 965 079–419 997 875 from Morex reference sequence with variants detected by mapping reads from Bowman against Morex reference in the aligned region.

**Table 1.** List of barley accessions included in BarleyVarDB.

No.	Accession	Abbreviation	Classify	Data_origin
1	AC_Metcalfe	AC	Australia cultivar	PRJNA324520
2	Baudin	BD	Australia cultivar	PRJNA324520
3	Commander	Commander	Australia cultivar	PRJNA324520
4	SFS2.1	EC2.1	EC wild barley	PRJNA324520
5	SFS2.2	EC2.2	EC wild barley	PRJNA324520
6	SFS7.1	EC7.1	EC wild barley	PRJNA324520
7	SFS7.2	EC7.2	EC wild barley	PRJNA324520
8	Fleet	Fleet	Australia	PRJNA324520
9	Hindmash	Hindmash	Australia cultivar	PRJNA324520
10	La_Trobe	La_Trobe	Australia cultivar	PRJNA324520
11	Scope	Scope	Australia cultivar	PRJNA324520
12	Vlamingh	Vlamingh	Australia cultivar	PRJNA324520
13	W1	W1	Tibetan wild barley	PRJNA324520
14	WI4304	WI4304	Australia breeding line	PRJNA324520
15	X1	X1	Tibetan wild barley	PRJNA324520
16	Barke	Barke	German cultivar	PRJEB628
17	Bowman	Bowman	US cultivar	PRJEB628
18	Haruna_Nijo	Haruna_Nijo	Japan cultivar	PRJEB628
19	Igri	Igri	Japan cultivar	PRJEB628
20	B1k–04–12	B1k–04–12	wild barley	PRJEB628
21	Morex	Morex	US cultivar	Barley Reference

## Database utility description

### Search SNP/InDel/SSR by Id

In this interface, web users can retrieve certain SNP/InDel/SSR by entering their identifiers (such as ‘SNP|chr-1H|000000749’, ‘IND|chr1H|000023712’, ‘SSR|chr1H|-000068174’), which is unique in the database (Figure 2c). Two pieces of information are included in the identifier: variation type (‘SNP’, ‘IND’, ‘SSR’) and chromosome coordinate (e.g. chr1H|000000749). For example, ‘SNP|chr1H|00-0000749’ means a SNP at 749 bp in chromosome 1H of barley pseudo-molecular reference. Generally, there are four types of genotype information given in the queried result as (‘./.’, ‘0/0’, ‘0/1’, ‘1/1’). Among them, ‘0/0’ represents missing data for polymorphic SNPs/InDels position in certain sample, and ‘0/0’ for homozygous genotype of reference allele, ‘0/1’ for heterozygous genotype, and ‘1/1’ for homozygous genotype of the alternative allele.

### Search SNP/InDel/SSR in target region

In this interface, a target region together with barley accessions can be given and used to retrieve SNP/InDel information (Figure 2d). Then the SNP and InDel Information in the target region among the given accessions can be obtained. For SSR search, a certain region can also be given to acquire SSR records in the target regions.

### Search SNP/InDel/SSR within gene

Genomic variants in gene region are quite useful, because different allele of them may result in the change of phenotype and they can be used as a functional marker to track target genes of agronomic importance. The genetic effects of each SNP or InDel were assessed based on their locations in gene structure (5ʹUTR, exon, intron, 3ʹUTR) and the change of gene coding products (nonsense mutation, missense mutation, open reading frame shift and so on) using snpEff (30). Because the high coverage of whole genome sequencing, the information of these SNP/InDels location and genetic effects will also be conductive to predict the candidate genes in a target region.

### Search polymorphic SNP/InDel between two samples

Comparison of genetic background between two parent lines of a mapped population is a common task for genetic research. In this interface, the user can directly get SNP/InDel sites with different alleles for two given samples (Figure 2a,b). This information is very useful in developing polymorphic molecular markers for agronomic QTL mapping or marker-assistance selection breeding. PCR primers to examine polymorphic InDels can be automatically designed after selecting the hyperlinked InDel names. For polymorphic SNP sites, they can be converted to CAPS markers for easily detection through PCR and electrophoresis if they are located in the recognizing sequence of restriction enzymes. Recognizing sequence analysis and primer design that are both performed by a background processing script except you should choose the type of predicted restricted enzymes you prefer for primer design.

### Web-based tools for barley genetic researchers

**Figure 3.**
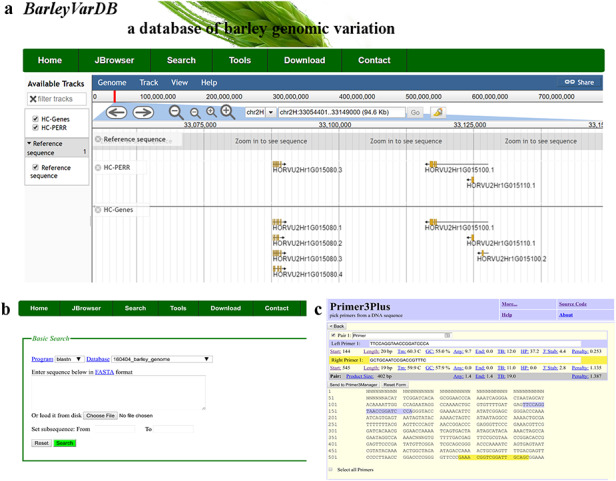
Examples of software applications in BarleyVarDB. (a) Example of gene annotations of barley genome reference displayed in JBrowse. (b) Search interface of barley reference genome blast server. (c) Example of primer designs in barley using primer3plus.

JavaScript-based Genome browser (JBrowse) (31) is a user-friendly and interactive web interface for displaying and manipulating whole genome-wide datasets such as reference genome sequence, reference annotation information, genomic variations, gene expression levels and so on. All these large sizes of dataset are stored in structure database, which will accelerate the process of retrieving and displaying (Figure 3a). Meanwhile, a BLAST server for the latest version of barley genome reference is set up in this website (Figure 3b). The pre-build BLAST databases included whole genome sequence, coding sequences, transcript sequences and predicted protein sequences of barley reference. Besides, a web-based PCR primer design system is also implemented with primer3plus as the core program (Figure 3c). Except the normal primer design function, some special features for barley genome are added. For example, users can directly primers by enter the chromosome coordinate of the target region in barley. It will save barley genetic researchers a lot of time and increase the efficiency of their research.

## Future improvement

As more barley germplasms are sequenced, we will continuously capture and share genomic variation information from more sequenced data and add their genomic variation information into BarleyVarDB to provide a comprehensive database for barley genomic variation. Meanwhile, more web-based software resources for the barley genetic research community to explore barley genomic data will be developed and incorporated into BarleyVarDB. Feedbacks from users are welcome and will inform future developments and updates of BarleyVarDB.
